# Conducted Vasoreactivity: the Dynamical Point of View

**DOI:** 10.1007/s11538-014-0058-0

**Published:** 2015-01-13

**Authors:** D. E. Postnov, A. Y. Neganova, O. V. Sosnovtseva, N.-H. Holstein-Rathlou, J. C. Brings Jacobsen

**Affiliations:** 1 Department of Physics, Saratov State University, Astrakhanskaya Str. 83, Saratov, 410026 Russia; 2 Department of Biomedical Sciences, Copenhagen University, Blegdamsvej 3, 2200 Copenhagen N, Denmark

**Keywords:** Endothelial cell, Conducted vasodilation, Membrane potential, Bistability

## Abstract

Conducted vasodilation is part of the physiological response to increasing metabolic demand of the tissue. Similar responses can be elicited by focal electrical or chemical stimulation. Some evidence suggests an endothelial pathway for nondecremental transmission of hyperpolarizing pulses. However, the underlying mechanisms are debated. Here, we focus on dynamical aspects of the problem hypothesizing the existence of a bistability-powered mechanism for regenerative pulse transmission along the endothelium. Bistability implies that the cell can have two different stable resting potentials and can switch between those states following an appropriate stimulus. Bistability is possible if the current–voltage curve is *N* shaped instead of monotonically increasing. Specifically, the presence of an inwardly rectifying potassium current may provide the endothelial cell with such properties. We provide a theoretical analysis as well as numerical simulations of both single- and multiunit bistable systems mimicking endothelial cells to investigate the self-consistence and stability of the proposed mechanism. We find that the individual cell may switch readily between two stable potentials. An array of coupled cells, however, as found in the vascular wall, requires a certain adaptation of the membrane currents after a switch, in order to switch back. Although the formulation is generic, we suggest a combination of specific membrane currents that could underlie the phenomenon.

## Introduction

In the arteriolar bed, local application of vasoactive agents or current elicits local diameter changes that spread up- and downstream along the vascular wall and eventually may spread into adjacent vessels (Gustafsson and Nolstein-Rathlou 1999; Bagher and Segal 2011; Hill 2012; Segal 1991; Emerson and Segal 2001; Emerson et al. 2002; Wit et al. 2000; Figueroa et al. 2003, 2007; Dora et al. 2003). The first observations of such remote effects of localized stimulation were made nearly 90 years ago by Krogh et al. (1922), while more detailed studies of such vascular conducted responses (VCRs) only started in 1970s (Duling and Berne 1970). The VCR is believed to play a central role in the local regulation of blood flow within a tissue. If local metabolism of a tissue changes due to changes in activity, the vessels of the upstream arteriolar network must change resistance in a coordinated manner to continuously match perfusion to demand. In this process, the VCR is believed to be critical by conveying information upstream to more proximally located feeding arterioles (Bagher and Segal 2011). Vasodilator signals generated in the endothelium can propagate over significant distances (several millimeters) along the vessel. In this process, gap junction coupling between the cells of the vascular wall appears to be crucial (Wit et al. 2000; Figueroa et al. 2003). Together with an apparently high speed of propagation (more than 20 mm/s Emerson et al. 2002; Stevens et al. 2000; Tsuchiya and Takei 1990), this points to an electrical nature of the phenomenon.

While the change in vessel diameter can decay with the distance from stimulation site in many cases (Segal 1991; Emerson et al. 2002), it appears to be nondecaying in other cases implying the involvement of some kind of regenerative process (Emerson and Segal 2001; Figueroa et al. 2003, 2007). However, since generally endothelial cells are considered to be electrically unexcitable (Nilius and Droogmans 2001), lacking the spiking characteristics typical for excitable cells, a regenerative process in the traditional sense with threshold characteristics and an all-or-none response seems unlikely to underlie nondecaying VCR.

Hypothesized pathways for regenerative propagation of vasodilatory signals remain controversial due to complex response of the endothelial cell to different stimuli and due to high variability of endothelial cells depending on the specific vascular bed from which they originate. Figueroa et al. (2007) suggested a neuron-like mechanism based on the existence and functionality of voltage-gated sodium channels. This mechanism implies a calcium rise in EC, as it has been observed in some cases (Zhang et al. 2006). Rivers (1997) suggested that inwardly rectifying potassium channels ($K_{ir}$) cause direct spread of hyperpolarization along the endothelium and subsequent spread of the signal to the smooth muscle cell layer. This hypothesis was later supported by Crane et al. (2004). On the other hand, a recent review by Hill (2012) suggested that a true regenerative response may in fact not be needed and that experimental data can be explained on the basis of a nonlinear relation between the strength of stimulatory signal and the resulting mechanical response of the SMC.

Taken together, it remains unclear what underlies the frequently observed apparently nondecaying spread of vasodilation in some vascular beds.

### Previous Modeling Studies of EC Dynamics

A number of modeling studies have focused on spreading vascular responses in order to estimate conditions that are necessary to sustain decaying or nondecaying electrical signals along a vascular bed.

Hirst and Neild (1978) applied traditional cable theory modeling a vessel segment as a continuous wire. A similar approach was used for simulations of the spread of membrane potential changes in microvascular trees (Crane et al. 2004). Diep et al. (2005) developed a computational model of spreading vasodilation based on a simplified representation of both smooth muscle cells and endothelial cells. Kapela et al. (2010) suggested comprehensive multicellular model of vasoreactivity in rat mesenteric arterioles and focused on quantitative simulation of the spread of a decaying response. Hald et al. (2012) extended the latter model to include a more detailed microanatomy as well as intracellular diffusion and analyzed the spread of changes in membrane potential following electrical stimulation.

Although recently Hald et al. (2014) showed that under certain conditions the length constant of a decaying signal may appear infinite, these above models have not produced evidence of domains in which a regenerative component is present and the question of the existence of a complete or partially regenerative mechanism remains open.

### Regenerative Pulse Transmission and Whole-Cell Electric Properties

It is convenient to represent both single cells and the vessel wall in the form of equivalent electric circuits (Diep et al. 2005; Kapela et al. 2010). We use this approach to illustrate some basic features needed for regenerative pulse transmission. Our approach is based on the assumption that regenerative (nondecremental) pulse transmission is possible only when there is some energy supply provided by ECs. Speaking in terms of equivalent electric circuits, this implies the presence of *negative* whole-cell resistance (or conductance), provided by an $N$-shaped whole-cell current–voltage curve.

**Fig. 1:**
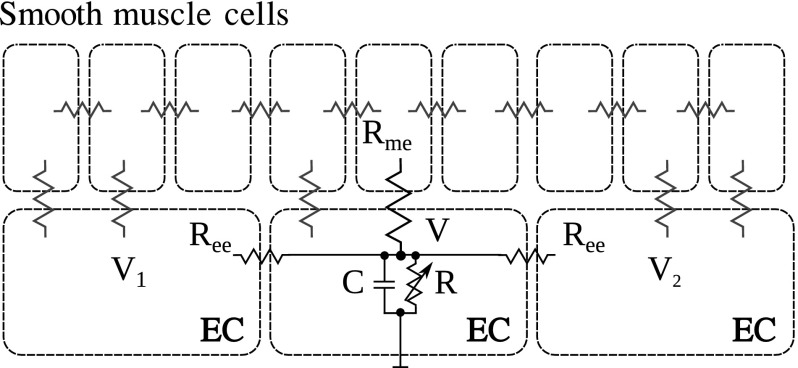
Schematic representation of coupled cells in the form of equivalent electric circuits. $R_\mathrm{ee}$ and $R_\mathrm{me}$ represent the resistances of gap junctions between endothelial cells and between endothelial cells and smooth muscle cells, respectively. $C$ and $R$ denote endothelial whole-cell capacitance and resistance, respectively. $V$, $V_1$, and $V_2$ denote the membrane potentials of the adjacent endothelial cells

In Fig. 1, a small fragment of a two-layer vascular wall is shown schematically. Here, an endothelial cell (EC) is coupled to two adjacent ECs and with some of the adjacent smooth muscle cells (SMCs).

The coupling between cells occur via endothelial gap junction with total resistance $R_\mathrm{ee}$ between two ECs and via myoendothelial gap junctions with total resistance $R_\mathrm{me}$ between one EC and all the SMCs, to which it is coupled. The EC itself is characterized by the whole-cell capacitance $C$ and whole-cell resistance $R$. The latter incorporates all membrane ionic currents (nonlinear and having their own kinetics) and, thus, can provide a complex shape of the whole-cell current–voltage curve. For the sake of simplicity, we neglect the current through myoendothelial gap junctions since the corresponding resistance $R_\mathrm{me}$ is much higher (probably several hundred times) than the endothelial–endothelial resistance $R_\mathrm{ee}$ (Diep et al. 2005).

The membrane potential $V$ of the central EC is governed by the following equation:

1 $$\begin{aligned} C \frac{\hbox {d}V}{\hbox {d}t}&= (V_1 -V)/R_\mathrm{ee} -V/R -(V-V_2)/R_\mathrm{ee}, \end{aligned}$$

where $V_1$ and $V_2$ are the membrane potentials at the two neighboring endothelial cells. At the equilibrium, one can write:

2 $$\begin{aligned} V_1+V_2 - 2V-\frac{R_\mathrm{ee}}{R}V&= 0. \end{aligned}$$

Suppose that, at time $t=0$, the voltage $V_1$ sharply rises (or drops) to some new value and the system quickly reaches a new equilibrium, while $V_2$ remains unchanged. Introducing $\varDelta V_1=V_1|_{t>0}-V_1|_{t<0}$ and the corresponding change $\varDelta V=V|_{t>0}-V|_{t<0}$, nondecremental pulse transmission implies that $\varDelta V \ge \varDelta V_1$. This can be satisfied if $(2+R_\mathrm{ee}/R) \le 1$, which immediately gives:

3 $$\begin{aligned} R+R_\mathrm{ee}&\le 0. \end{aligned}$$

The only way to satisfy this relation is to have negative $R$.

The notion of negative (true or differential) resistance is long known paradigm in physics to describe the processes in devices that supply energy to a signal http://en.wikibooks.org/wiki/Circuit_Idea/Revealing_the_Mystery_of_Negative_Impedance. In physically realizable systems, it comes in the form of *S*-shaped or *N*-shaped current–voltage curves (CVC). Note that such CVCs have been known for a long time for living cells (Fishman and Macey 1969).

### Bistability Hypothesis

If a two-component nonlinear system has an $N$-shaped CVC, then its dynamical features are mainly governed by the number and stability of equilibrium points. Namely, if there is a single and unstable equilibrium point, then the system shows self-sustained oscillations arising in the endothelium. If the equilibrium point is single and stable, then system can be excitable. If, with an $N$-shaped CVC and three equilibrium points, the system does not show excitability or self-sustained behavior, then the only possibility is bistability.

Vascular endothelial cells are known as nonexcitable (Nilius and Droogmans 2001), and there is no evidence of self-sustained oscillations. However, there is an experimental evidence of N-shaped CVC’s by Voets et al. (1996) and a strong suggestion of bistability by Jiang et al. (2001) in the form of a bimodal distribution of resting potentials over the population of more than 700 cells. Additionally, Zhang et al. (2006) demonstrated that endothelial cells can remain in the depolarized state for a long period until the electrical stimulus is removed, which is not consistent with excitable dynamics.

All this allows us to hypothesize that bistable dynamics of the endothelial vascular layer might be an important component for the vasodilatory-conducted response. The rest of this paper is focused on investigating the consistency and reasonability of this hypothesis from the dynamical viewpoint. Specifically, we (i) suggest a minimal model for an EC response suitable for theoretical analysis, (ii) analyze whether selected model components are capable of producing bistable dynamics, (iii) check whether the single bistable unit can provide amplification of an external stimulus, and (iv) investigate under which conditions a discrete array of such units is capable of providing a nondecremental propagation of pulses of arbitrary duration. All the above points allow us to suggest a bistability-powered mechanism of conducted vasodilation, which is not in conflict with available data.

With this approach, we focus on a direct spread of hyperpolarization from the site of stimulation along the endothelium. According to this scenario, there is no substantial change in EC calcium concentration at remote sites (i.e., far from the stimulation site) reached by the hyperpolarization wave. Rather, the dilatatory response is caused by the direct spread of the hyperpolarization to the smooth muscle cell layer through myoendothelial gap junctions.

## The Model

Since the number, roles, and contributions of specific ionic currents in the EC are a matter of debate, we implement our model using a minimal number of functional components, each being a simplified and linearized description of some prototypical ionic current.

**Fig. 2:**
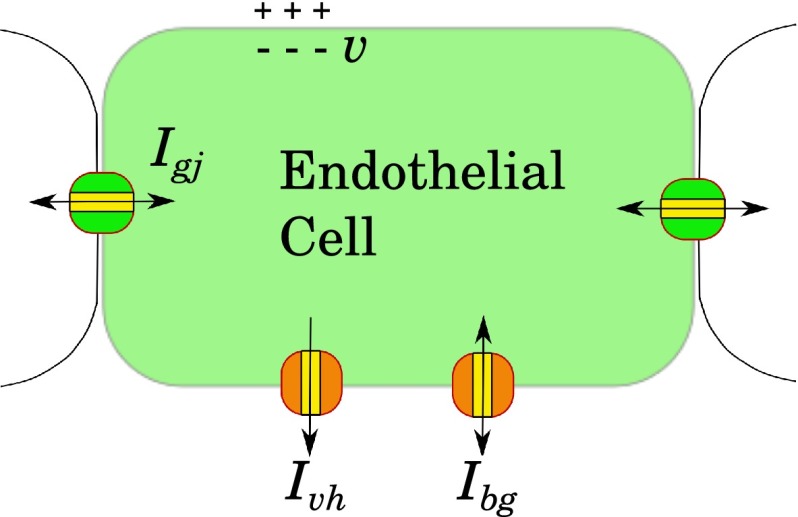
Simplified representation of an endothelial cell, providing the functional model Eqs. (4)–(9). All pathways and ionic currents that are not active at rest and are omitted in the hyperpolarized state. $I_\mathrm{gj}$—gap junction current, $I_\mathrm{vh}$—voltage-sensitive hyperpolarizing current and $I_\mathrm{bg}$—nonspecific background current

The cellular pathways that may contribute to the formation of a conducted response are discussed in numerous papers (Rivers 1997; Figueroa et al. 2007; Crane et al. 2004; Bagher and Segal 2011; Hill 2012). A considerable part of these mechanisms require cell depolarization. However, if we focus on conducted hyperpolarization and assume that all depolarization-activated currents are completely closed at the resting potential, then quite few dynamical components remain. We include just three currents in our minimal model, being the voltage-gated hyperpolarizing current, $I_\mathrm{vh}$, unspecific background current, $I_\mathrm{bg}$, and unspecific gap junction current, $I_\mathrm{gj}$ (Fig. 2). With this assumption, the membrane potential $v$ is governed by the following equations:

4 $$\begin{aligned} C \frac{\hbox {d}v}{\hbox {d}t}&= - I_\mathrm{vh} - I_\mathrm{bg} + I_\mathrm{gj},\end{aligned}$$

5 $$\begin{aligned} I_\mathrm{vh}&= g_\mathrm{vh}w(v-v_\mathrm{vh}),\end{aligned}$$

6 $$\begin{aligned} I_\mathrm{bg}&= g_\mathrm{bg}(v-v_\mathrm{bg}),\end{aligned}$$

7 $$\begin{aligned} I_\mathrm{gj}&= g_\mathrm{gj}\left( \sum _j^n v_ j -v_\mathrm{bg}\right) ,\end{aligned}$$

8 $$\begin{aligned} \tau _\mathrm{vh}\frac{\hbox {d}w}{\hbox {d}t}&= w_{\infty }(v)-w,\end{aligned}$$

9 $$\begin{aligned} w_{\infty }(v)&= \frac{1}{2\delta }(\delta +|v-h|-|v-h+\delta |). \end{aligned}$$

$C$ is the whole-cell capacitance, and $I_\mathrm{vh}$, $I_\mathrm{bg}$ and $I_\mathrm{gj}$ are ionic currents. In (7), $j$ counts the $n$ neighbors of the cell under consideration. In Eq. (5), $w$ is the gating variable governed by the Eq. (9). A prototype for voltage-gated hyperpolarizing current $I_\mathrm{vh}$ is the inwardly rectifying potassium current (Nilius and Droogmans 2001; Crane et al. 2004). Expression (9) describes the piecewise linear activation function $w_{\infty }(v)$ that is governed by two parameters $h$ and $\delta$. $h$ describes the membrane potential above which ion channels completely closed and $\delta$ describes the membrane potential range between completely open and completely closed states.

This simplified description implies that the population of ion channels can be:

(i) completely closed if $v> h$ since $w_{\infty }(v)=0$,
(ii) completely open if $v<(h-\delta )$, since $w_{\infty }(v)=1$, and
(iii) open with a fraction linearly proportional to the membrane potential if $(h-\delta )<v<h$.

**Fig. 3:**
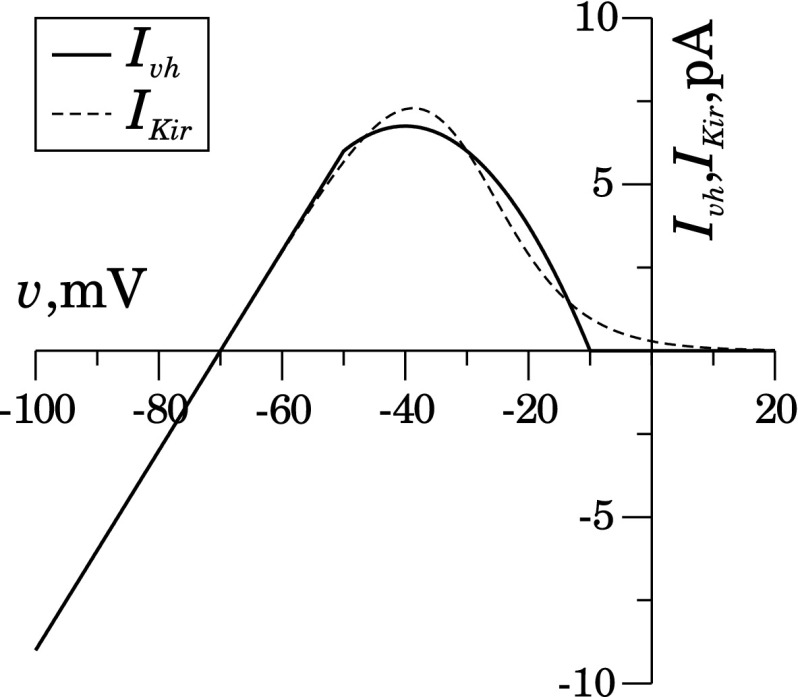
Model representation of the voltage-gated hyperpolarizing current $I_\mathrm{vh}$ as an inwardly rectifying potassium current. *Solid curve* is calculated from Eqs. (5), (8), and (9) ($h=-10.0$ mV, $\delta =40.0$ mV, $v_\mathrm{vh}=-70$ mV and $g_\mathrm{vh}=0.3$ nS). *Dashed curve* is the data-fitted exponential approximation from Ref. Silva et al. (2007) using $v_K=-70$ mV and $g_{Kir}=0.3$ nS

With the selection of parameters in the range of $v_\mathrm{vh}\in$ $[-70$; $-90$ mV], $g_\mathrm{vh}\in ~[0.25$ nS; $0.35$ nS], $h\in$ $[-20$; $-10$ mV], and $\delta \in ~$[30; 40 mV], the resulting current–voltage plot for this current reproduces well the typical features of the inwardly rectifying potassium channel (Silva et al. 2007; Crane et al. 2003; Yang et al. 2003). The reversal point will be at the Nernst potential for potassium, the maximal outward current will be of order of $10$ pA and reached in the range of $v\in [-40$; $-20$ mV], and the current vanishes at $v$ more positive than [$-20$;$-10$ mV]. In Fig. 3, the solid curve is calculated according Eqs. (5), (8), and (9), while the dashed curve shows data-fitted exponential approximation used in Silva et al. (2007).

## Single-Cell Bistability

In this section, we investigate under which conditions the endothelial cell can show bistable behavior. It can occur if total-cell (tc) current–voltage curve has three zeroes at $v_1 < v_2 <v_3$. This leads to bistability in the form of two coexisting stable resting states at $v_1$ and $v_3$ and provides a segment with negative resistance (conductance) near the $v_2$.

First, we consider either an isolated cell or a population of cells with equal resting voltage, i.e., gap junction current vanishes. The total-cell current is as follows:

10 $$\begin{aligned} I_\mathrm{tc}&= I_\mathrm{vh} + I_\mathrm{bg}. \end{aligned}$$

Second, assuming fast activation of $I_\mathrm{vh}$, according to (8), Spector et al. (1996) one can write:

11 $$\begin{aligned} I_\mathrm{tc}&= g_\mathrm{vh}(v-v_\mathrm{vh})w_{\infty }(v) + g_\mathrm{bg}(v-v_\mathrm{bg}), \end{aligned}$$

where $w_{\infty }(v)$ is defined by (9) ($v$ omitted hereafter). In order to find points of minimum and maximum, consider the derivative

12 $$\begin{aligned} \frac{\hbox {d}I_\mathrm{tc}}{\hbox {d}v}&= g_\mathrm{vh} ((v-v_\mathrm{vh})\frac{\hbox {d}w_{\infty }}{\hbox {d}v} +w_{\infty }) + g_\mathrm{bg}. \end{aligned}$$

**Case 1** If $v > h$, $w_{\infty }=0$, and $\frac{\hbox {d}w_{\infty }}{\hbox {d}v}=0$ (the channel is completely closed) and

13 $$\begin{aligned} \frac{\hbox {d}I_\mathrm{tc}}{\hbox {d}v}&= g_\mathrm{bg}. \end{aligned}$$

Thus, at nonzero $g_\mathrm{bg}$, there are no extrema at $v > h$.

**Case 2** If $v < h-\delta$, $w_{\infty }=1$, and $\frac{\hbox {d}w_{\infty }}{\hbox {d}v}=0$ (the channel is completely open) and

14 $$\begin{aligned} \frac{\hbox {d}I_\mathrm{tc}}{\hbox {d}v}&= g_\mathrm{vh}+g_\mathrm{bg}. \end{aligned}$$

Again, since we assume that both $g_\mathrm{bg}$ and $g_\mathrm{vh}$ are positive, there is no minimum or maximum at membrane potential lower than $h-\delta$.

**Case 3** If $h-\delta < v < h$, the channel is open with a probability linearly proportional to the membrane potential and $w_{\infty }=(h-v)/\delta$, and $\frac{\hbox {d}w_{\infty }}{\hbox {d}v}=-1/\delta$. In that case, after some algebraic calculations, we have a single zero point for $\frac{\hbox {d}I_\mathrm{tc}}{\hbox {d}v}$ at:

15 $$\begin{aligned} v_1&= \frac{1}{2}(\delta (g_\mathrm{bg}/g_\mathrm{vh}) +h + v_\mathrm{vh}). \end{aligned}$$

This extremum exists if the condition $h-\delta < v_1 < h$ is maintained; hence,

16 $$\begin{aligned} -2\delta <\delta g_\mathrm{bg}/g_\mathrm{vh}+ v_{vh} -h < 0. \end{aligned}$$

Since we are looking for conditions for an $N$-shaped current–voltage curve, we expect two zero points for $\hbox {d}I_\mathrm{tc}/\hbox {d}v$. However, due to piecewise nature of $w_{\infty }$, the second of these points is at the discontinuity of $\hbox {d}w_{\infty }/\hbox {d}v$. Note that

17 $$\begin{aligned} \frac{\hbox {d}I_\mathrm{tc}}{\hbox {d}v}|_{v=h-0}&= -g_\mathrm{vh}(h-v_\mathrm{vh})/\delta + g_\mathrm{bg} <0,\; if\;\; \delta \frac{g_\mathrm{bg}}{g_\mathrm{vh}} <(h-v_\mathrm{vh}),\end{aligned}$$

18 $$\begin{aligned} \frac{\hbox {d}I_\mathrm{tc}}{\hbox {d}v}|_{v=h+0}&= g_\mathrm{bg} >0. \end{aligned}$$

If the condition (17) is satisfied, then $dI_\mathrm{tc}/dv$ changes sign at $v_2=h$, and hence, this is the second extremum. The existence of both $v_1$ and $v_2$ means that a plot of $I_\mathrm{tc}(v)$ is $N$-shaped.

The condition for bistability is as follows:

19 $$\begin{aligned} I_\mathrm{tc}(v_1)&> 0, \;\; I_\mathrm{tc}(v_2)&< 0. \end{aligned}$$

After some algebraic calculations, we get:

20 $$\begin{aligned} \left( \frac{\delta g_\mathrm{bg}}{g_\mathrm{vh}}+h-v_\mathrm{vh}\right) ^2&> 4 \frac{\delta g_\mathrm{bg}}{g_\mathrm{vh}} (v_\mathrm{bg} - v_\mathrm{vh})\end{aligned}$$

21 $$\begin{aligned} v_\mathrm{bg}>h. \end{aligned}$$

To summarize the results of analytical calculations, the model Eqs. (4)–(9) describe a bistable system that has two coexisting stable resting states if all four conditions (16), (17), (19), and (20) are satisfied. Note if (16) is true, then (17) is satisfied automatically.

**Fig. 4:**
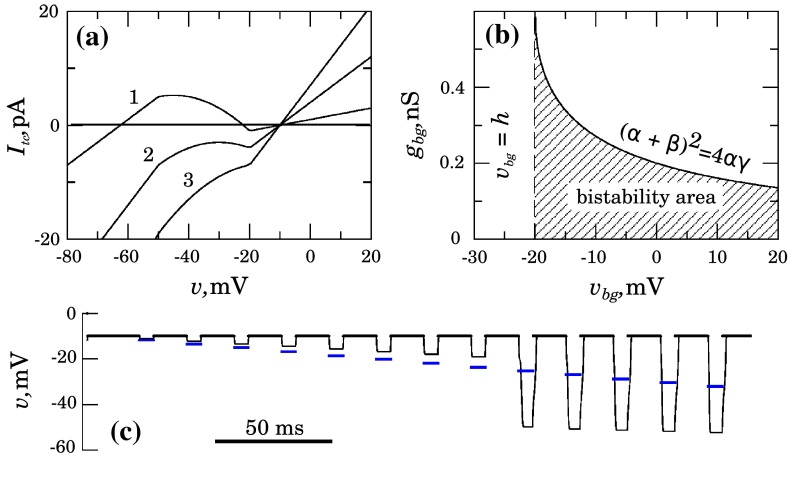
(Color figure online) Hyperpolarization-induced bistability. **a** Whole-cell $I-V$ curves at $v_\mathrm{bg}=-10$ mV, $h=-20.0$ mV, $\delta =30.0$ mV, $v_\mathrm{vh}=-80$ mV and $g_\mathrm{vh}=0.3$ nS. Curves 1, 2, and 3 correspond to $g_\mathrm{bg}$ = 0.1, 0.4, and 0.7 nS, respectively; **b** Estimated area of bistability on the ($v_\mathrm{bg}$, $g_\mathrm{bg}$) parameter plane is *shaded gray*; **c** The numerically simulated response on an external hyperpolarizing stimulation (*blue*) of increasing amplitude

The expressions above can be further simplified if one denotes: $\alpha =g_\mathrm{bg}/g_\mathrm{vh}$, $\beta =(h-v_\mathrm{vh})/\delta$, $\gamma =(v_\mathrm{bg}-v_\mathrm{vh})/\delta$. With this, finally, we have:

22 $$\begin{aligned}&\alpha < \beta ,\end{aligned}$$

23 $$\begin{aligned}&\alpha > \beta -2,\end{aligned}$$

24 $$\begin{aligned}&(\alpha +\beta )^2 > 4\alpha \gamma . \end{aligned}$$

where $\alpha$ describes the ratio between conductances of the two currents, while $\beta$ is the relative width (i.e., voltage range) of the partially open state of $I_\mathrm{vh}$.

How are strict these conditions? Inspection shows that they are satisfied within a physiologically reasonable parameter range (in particular, in the range outlined in the text of the model section). In other words, our model shows bistability if (i) the reversal voltage for the background current is more positive than the outward segment of the hyperpolarizing current $I_\mathrm{vh}$ and (ii) the conductance of the background current is within some specified (not narrow) range.

Figure  4 illustrates the above results at the specific choice of parameters: $v_\mathrm{bg}=-10$ mV, $h=-20.0$ mV, $\delta =30.0$ mV, $v_\mathrm{vh}=-80$ mV, so $\beta =2$, and $g_\mathrm{vh}=0.3$ nS. The panel (a) illustrates the changes in whole-cell current with increasing $g_\mathrm{bg}$. One can see that at $g_\mathrm{bg}=0.1$ nS [curve 1, all conditions (22)–(24) are satisfied], the $I_\mathrm{tc}(v)$ curve is $N$-shaped and has three zeroes. This is the case we associate with bistability. At $g_\mathrm{bg}=0.4$ nS [curve 2, (22) and (23) are true, but (24) is not] there is still a segment with negative slope, but the curve has a single zero point; thus, there is only one resting state at $v=-10$ mV. At $g_\mathrm{bg}=0.7$ nS [curve 3, (22) and (24) are not true], there are no minima or maxima on the total-cell current–voltage curve, and the hyperpolarizing current does not considerably affect the behavior caused by the background current.

The panel (b) shows the bistability zone on the plane of parameters of the background current $v_\mathrm{bg}$ and $g_\mathrm{bg}$. The upper limit of this area is determined by Eq. (24), while the lower limit is given by $g_\mathrm{bg}=0$. One can see that the maximal value of $g_\mathrm{bg}$ becomes lower while for large $v_\mathrm{bg}$, it still has a considerable range, and thus, the regime of bistability is one of the main operating regimes of the model.

In order to confirm theoretical predictions, we simulate an external excitation of a single cell by rectangular pulses of increasing strength. We assume nonzero gap junction conductance and use some appropriate value for the term $\sum _j^n v_ j$ in (7) during hyperpolarizing pulses each of 10 ms length, which is sufficient for the model to reach a new equilibrium state. The panel (c) in Fig. 4 illustrates the results of a numerical simulation using the same set of parameter values, $g_\mathrm{bg}=0.1$ and $g_\mathrm{gj}=0.2$ nS. $v_j$ periodically takes either the value of the $v$ at the more positive of two resting states, $v_ j=v=v_\mathrm{bg}$ (so the coupling current vanishes), or an increasingly negative value $[-10$; $-27$ mV]. This is seen as blue horizontal segments. The response of the model is shown as black solid line. One can observe that the first 8 negative pulses cause a response that is smaller than the stimulus. However, at stimulus value $\approx$23 mV, the response amplitude increases drastically and reaches a value more negative than 50 mV. We can relate this behavior to possible regenerative pulse transmission by an array of endothelial units.

## Regenerative Pulse Transmission: an Array of Bistable Elements

**Fig. 5:**
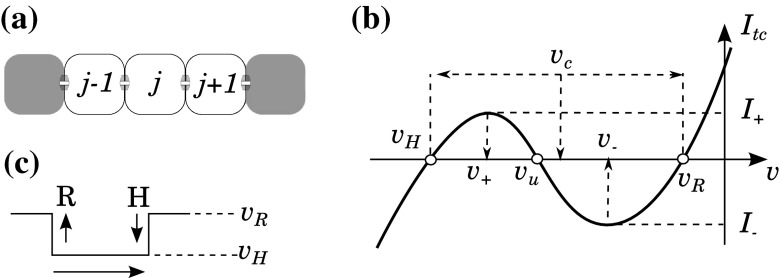
On propagation of a hyperpolarizing pulse along an array of one-component bistable units. **a** An array of bistable elements. **b** Hyperpolarizing pulse traveling from left to right (from the $(j-1)$-th unit toward the $(j+1)$-th unit). $H$ and $R$ denote the hyperpolarizing and resting fronts, respectively. **c** The schematic current–voltage curve and the equilibrium points for the single bistable unit

The bistable features of the single unit discussed in the previous section are crucial for the hypothesized mechanism of regenerative pulse transmission, but they are not sufficient to provide the expected behavior. An array of bistable units can behave differently in comparison with the behavior of an individual unit. In this section, we define conditions for a hyperpolarizing pulse of arbitrary duration conducted along an array of model units (Fig. 5a, b). This means that an array of coupled EC’s should support a propagated transition to a stable hyperpolarized state, and then, possibly after some recovery, it should equally support a propagated transition back to the initial resting state. We denote these transitions as well as the corresponding spatiotemporal patterns with letters H (hyperpolarized) and R (resting), respectively.

Similar problems of propagating bistable fronts has been addressed in physics with respect to spatiotemporal dynamics of reaction–diffusion systems, and the so-called nonequilibrium Ising–Bloch bifurcation (Coullet et al. 1990; Hagberg and Meron 1994). A vascular bed is best approximated by an array or grid of discrete bistable units but not by a continuous medium. Thus, our description is related to coupled ordinary differential equations (ODEs), rather than partial differential equations (PDEs). In such systems, there is a parameter region where front fails to propagate due to discrete description of the media (Keener 1987; Laplante and Erneux 1992; Hagberg and Meron 1998). Recently, relevant results were published using bistable FitzHugh–Nagumo (FHN) model (Müller et al. 2013).

The main findings can be summarized as follows: (i) there is a critical coupling strength that defines a parameter range, where propagation of bistable fronts is supported; (ii) in an array of one-component bistable units^1^, only one type of transition (in our notations, H or R) can propagate due to asymmetry of coexisting states; (iii) in an array of two-component bistable units with symmetric coexisting states, the propagation of both types of transition is possible. In FHN model, this is due to the dynamics of the inhibitor (slow variable) that provides an overshoot during transition of the unit to a new stable state.

Let us explain some of the above results.

Suppose, we have an array of bistable units coupled as shown in Fig. 5a. If the coupling is weak enough, we can consider interaction only with nearest neighbors. For $j$-th unit, one can write:

25 $$\begin{aligned} \frac{\hbox {d}v}{\hbox {d}t}C=-I_\mathrm{tc} + g_\mathrm{gj}( v_{j-1} + v_{j+1} - 2v_j). \end{aligned}$$

Assume that $C$ = 1.0. Here, the *N*-shaped $I_\mathrm{tc}$ has three equilibrium points: $v_\mathrm{R}$ at rest, $v_\mathrm{H}$ at the hyperpolarized state, and an unstable state, $v_\mathrm{u}$, somewhere in between. The coupling term provides the driving force for switching, depending on the states of the $(j-1)$-th and the $(j+1)$-th units. If all units of the array are in the same state (either $v_\mathrm{R}$ or $v_\mathrm{H}$), then coupling current vanishes. If, say, the left part of the array, including the $(j-1)$-th unit, is in the $v_\mathrm{H}$ state, but the right part of array, including the $(j+1)$-th unit is in the resting state $v_R$, then the driving force $F$ is

26 $$\begin{aligned} F=2g_\mathrm{gj}( v_\mathrm{c} - v_ j), \end{aligned}$$

where $v_\mathrm{c}=(v_\mathrm{R}+v_\mathrm{H})/2$. Note that

- In order to provide an escape from the stable state, $F$ should be strong enough to drive the unit through the extrema $I_+$ or $I_-$, which means that $2g_\mathrm{gj}( v_\mathrm{c} - v_+)>I_+$ or $2g_\mathrm{gj}( v_\mathrm{c} - v_-)<I_-$. This relation gives the minimal value for $g_\mathrm{gj}$;
- At $v_j= v_c$, the driving force vanishes, and the behavior of $j$-th unit depends on the sign of $I_\mathrm{tc}(v_\mathrm{c})$. In turn, it is determined by the location of $v_u$. If $(v_\mathrm{u}-v_\mathrm{H})>(v_\mathrm{R}-v_\mathrm{u})$, then $v_\mathrm{u}>v_\mathrm{c}$ and $j$-th unit tends to be the depolarized state. In the opposite case, $v_\mathrm{u}<v_\mathrm{c}$, the $j$-th unit tends to the hyperpolarized state. In the completely symmetric case, $F$ vanishes at $v_\mathrm{u}=v_\mathrm{c}$ and the behavior is undetermined.

The above consideration means that the possibility of spatial spreading of a $H$-front, for example, is completely determined by parameters of individual model [Eqs. (4)–(9)]. More importantly, if conditions for switching by a $H$-front are satisfied, then, automatically, conditions for switching by a $R$-front are not. Thus, a sequential propagation of $H$ and $R$ fronts is not possible, and hence, the propagation of hyperpolarizing pulses along an array of one-component model units cannot be observed.

**Fig. 6:**
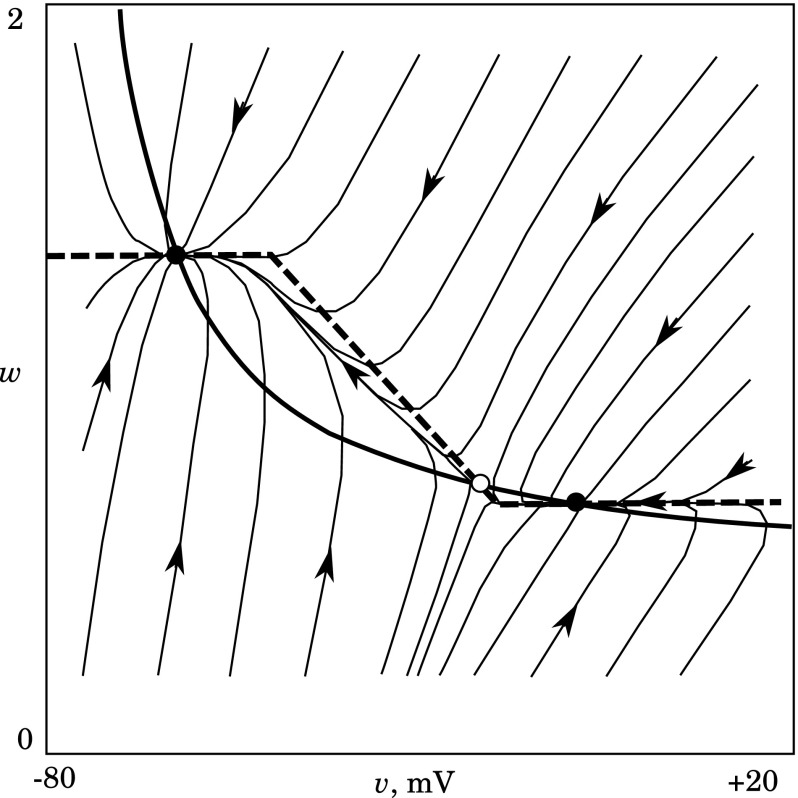
Nullclines and phase portrait for the model Eqs. (5)–(9) at $C=0.1$, $\tau _w=0.1$, $g_\mathrm{vh}=0.3$, $v_\mathrm{vh}=-80$, $g_\mathrm{bg}=0.1$, $v_\mathrm{bg}=-10$, $h_\mathrm{vh}=-20$, $\delta _\mathrm{vh}=30$. *Thick solid* and *dashed curves* represent $v-$nullcline and $w-$nullcline, respectively. *Thin curves with arrows* are the representative phase trajectories. *Filled* and *open circles* denote the stable and saddle equilibrium states, respectively

**Fig. 7:**
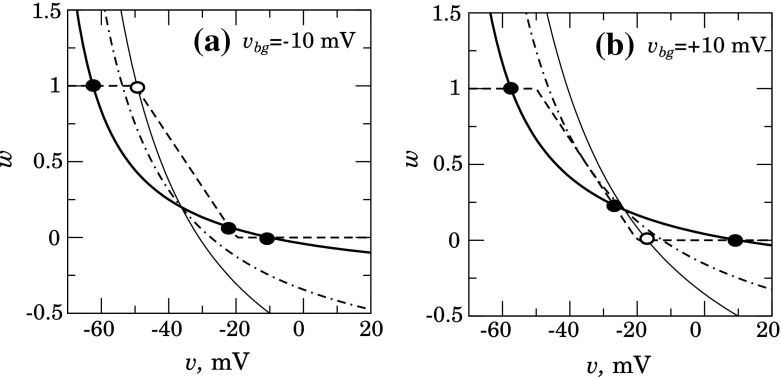
On the possibility of coupling-induced transition. The changes of $v$-nullcline position when $g_\mathrm{gj}=0.0$ nS (*thick solid line*), $g_\mathrm{gj}=0.05$ nS (*dash-dotted line*), and $g_\mathrm{gj}=0.1$ nS (*thin solid line*), for two different $v_\mathrm{bg}$ **a** $-$10 mV and **b** 20 mV

In Fig. 6, the model nullclines are shown together with the bunch of the representative phase trajectories. It is seen that the behavior of the single unit is essentially one-dimensional. Although the plot shows the case of $C=\tau _w$ (no fast-slow behavior), the structure of the phase portrait indicates that there will not be significant changes for the case of $C\ne \tau _w$.

Figure 7 shows nullclines intersection for different $v_\mathrm{bg}$ and for increasing $g_\mathrm{gj}$. The unit under consideration is assumed to be located between the two other units, one of which is in the resting state and the other is in the stable hyperpolarized state. It is clearly seen that strengthening of gap junctional coupling removes the bistable features, leaving only one point of intersection of nullcline. This describes the single stable state (open circle) with activated (a) or inactivated (b) voltage-gated hyperpolarizing current $I_\mathrm{vh}$.

To summarize, in spite of the bistable features of the individual unit, an array of gap junction coupled units described by Eqs. (4)–(9) seems to be unable to provide dynamical patterns associated with regenerative pulse transmission, which implies an equally possible propagation of both H- and R-fronts. Numerical simulations confirm the analysis presented above. Successful propagation of H-front was observed at the set of parameters also used for Fig. 7a, but all attempts to trigger reversal of the system by an R-front were unsuccessful.

## Regenerative Pulse Transmission: Extended Model

The results of the previous section show that our model is too minimalistic and lacks some essential functional components to simulate regenerative pulse transmission. The qualitative consideration of the mechanisms of propagation of bistable fronts suggests that we should add some mechanism, providing an adaptation of the bistable unit to the recently achieved state, thus facilitating the transition back. From a dynamical point of view, this means the introduction of a slow variable that could re-balance the system after the transition from one state to another has occured. It could be achieved by means of:

(i) Slow inactivation of the hyperpolarizing current (a slow decline of $g_\mathrm{vh}$);
(ii) Inactivation of the hyperpolarizing current by inclusion some additional $w$-independent part into $I_\mathrm{vh}$;
(iii) Slow activation of the background current (slow increase of $g_\mathrm{bg}$);
(iv) Activation of the background current by introducing some additional part.

Close inspection reveals that the variants (ii), (iii), and (iv) are essentially equivalent, since they can be transformed one into another by suitable adjustment of $g_\mathrm{bg}$ and $v_\mathrm{bg}$. Thus, we can limit our consideration to only two variants of the model extension. The model equations are as follows:

27 $$\begin{aligned} C \frac{\hbox {d}v}{\hbox {d}t}&= - (1-kx)I_\mathrm{vh} - (1+mx)I_\mathrm{bg} + I_\mathrm{gj},\end{aligned}$$

28 $$\begin{aligned} \tau _{x}\frac{\hbox {d}x}{\hbox {d}t}&= x_{\infty }(v)-x,\end{aligned}$$

29 $$\begin{aligned} x_{\infty }(v)&= \frac{1}{2\delta }(\delta +|v-h|-|v-h+\delta |), \end{aligned}$$

where the new gating variable $x$ with characteristic time $\tau _x \gg \tau _w$ is introduced. $I_\mathrm{vh}$, $I_\mathrm{bg}$, and $I_\mathrm{gj}$ are as described previously by Eqs. (5)–(9). Note that, for the sake of simplicity, we use the same step-like activation functions for both $w_{\infty }$ and $x_{\infty }$.

**Fig. 8:**
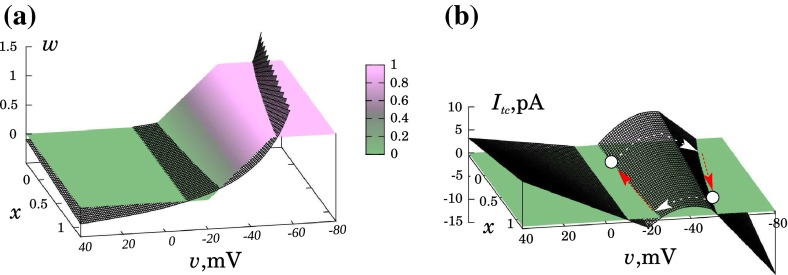
(Color figure online) **a** The nullclines and **b** the “voltage-clamped” total-cell current for the modified model. **a** $w$- and $x$-nullclines are given in gradient-colored surface, while $v$-nullcline is shown as a surface outlined by the set of *black lines*. **b** the *solid colored surface* indicates the zero level, while the surface outlined by the set of *black lines* represents the “voltage-clamped” total-cell current

Depending on the choice of the newly introduced parameters $k$ and $m$, we can simulate both $I_\mathrm{bg}$ additional activation ($k=0$, $m>0$) and $I_\mathrm{vh}$ inactivation ($k>0$, $m=0$). Figure 8 shows the nullcline surfaces (a) and a 3D plot of total-cell current (b) for $k=0$ and $m=0.4$. Since the nullclines for the $w$ and $x$ variables coincide, they are represented by the same gradient-shaded surface, while the $v$-nullcline surface is given by a set of black curves. One can observe that, for $x$ increasing from 0 to 1, the intersections of nullcline surfaces come close. The plot for the total-cell current shows a clear reduction of its maximum, thus facilitating the back transition of the system from the hyperpolarized state (both states as well as transitions between them are presented by open circles and arrows). All this confirms the proposition that the extended model should be able to show expected behavior.

## Simulation Results

In order to confirm the predictions made and to study the model behavior, numerical simulations are performed on an array of 60 coupled units for different combinations of control parameters. Note that the set of core model parameters has the same values as described in Sect. 2, while the extended model parameters $k$, $m$, $\tau _x$, and the duration of the hyperpolarizing stimulus $\varDelta t_\mathrm{stim}$ are varied in order to observe different propagation patterns.

**Fig. 9:**
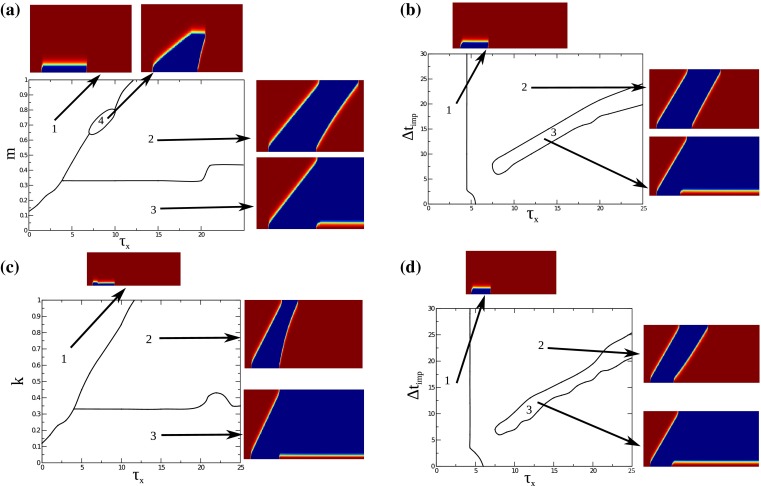
(Color figure online) Simulation results on the propagation of a hyperpolarizing pulse along an array of bistable units according (27)–(29). **a**, **b** Maps of regimes for the case of a slow additional activation of the background current **a** on the plane $(\tau _x, m)$ at $k=0$ and $\varDelta t_\mathrm{imp}=10$ and **b** on the plane $(\tau _x,\varDelta t_\mathrm{imp}=10)$ at $k=0$ and $m=$ $0.4$. **c**, **d** Maps of regimes for the case of slow inactivation of the hyperpolarizing current **c** on the plane $(\tau _x, k)$ at $m=0$ and $\varDelta t_\mathrm{imp}=10$, and **d** on the plane $(\tau _x,\varDelta t_\mathrm{imp}=10)$ at $k=0.4$ and $m=0$. Inserts show the wave propagation patterns that are representative for the corresponding segment of diagram. *Blue* and *red colors* encode the units in the hyperpolarized state and in the resting state, respectively. The *x*-axis in the inserts is time. The *y*-axis in the inserts corresponds to the position along the 60-units array

The simulation results are presented as color-coded diagrams (Fig. 9), where x axis is time, while the number of units within the array increases along the vertical axis. The red and blue colors correspond to the resting and hyperpolarized state of the unit, respectively. The numbers in the maps (a–d) indicate different propagation patterns shown in the inserts.

Let us describe first what happens in the area labeled (3) in all diagrams. The state near the bottom line of the diagram (a) and (c) corresponds to the vanishing both $k$ and $m$ and shows the behavior previously observed in two-component version of the model. Namely, the H-front of hyperpolarization successfully propagates, while the R-front triggered stops after $\varDelta t_\mathrm{stim}$ at second or third unit of the array and does not propagate any further. Such behavior can be observed up to some critical value of $k=0.33$ or $m$ $\approx 0.33$. What is observed at higher values of those parameters depends on $\tau _x$ describing how slow the adaptation is.

In the area labeled (1), if $\tau _x <4$, then the increasing of both $k$ and $m$ blocks the propagation of the H-front, which stops at some (parameter dependent) distance from the initial point and even moves back in some cases [top insert in the panel (c)]. This can be explained by the fact that adaptation does not only facilitate the backward (R) transition, but also complicates the forward (H) transition, if the adaptation develops too fast.

The area labeled (2) corresponds to the successful propagation of both H- and R-fronts. This is a dynamical image of the hypothesized regenerative pulse transmission: Irrespective of the specific duration of the hyperpolarizing pulse, it is transmitted along an array of bistable units. Note that, since the propagation speed for the H- and R-fronts may be not equal, the original duration $\varDelta t_\mathrm{stim}$ of the hyperpolarizing pulse is not maintained along the array. In the inserts for the panels (a) and (c), the R-front moves faster, so the duration of hyperpolarized state decreases while one moves away from the stimulus point.

A small area labeled (4) shows a specific case when the R-front catches the H-front and, thus, terminates the propagated hyperpolarization. Note the flat top of the hyperpolarization ”tongue.“ It indicates that the H-front completely stops before annihilation with R-front. Interestingly, the panels (b) and (d) indicate that close matching of $\varDelta t_\mathrm{imp}$ and $\tau _x$ prevents the propagation of R-front, at least for values of $k$ and $m$ selected for these plots.

The simulation results described above confirm the ideas and propositions of the extended model, demonstrating the applicability of the hypothesized bistability-based mechanism as a possible mechanism of conducted vasodilation.

## Discussion

Throughout our work, we addressed the possible dynamical (rather than physiological) mechanisms that may underlie the phenomenon of conducted vasodilation. Our study was inspired by the existence of controversial hypotheses and incomplete knowledge of specific ionic currents that could contribute to this phenomenon.

We showed that a simple model that incorporates (i) unspecified background current and (ii) a voltage-gated hyperpolarizing current, representing an inwardly rectifying potassium current, is able to reproduce the total-cell current–voltage curves obtained experimentally (Voets et al. 1996) and to show bistable behavior consistent with experimental observations (Jiang et al. 2001).

Note that for the sake of simplicity, electrical coupling to the SMC layer through myoendothelial junctions, necessary to cause SMC hyperpolarization and hence vasodilatation, was not included in the theoretical analysis. We performed, however, the simulation test if the proposed mechanism was stable under a relatively strong coupling to the SMC layer. Such simulations showed that myoendothelial coupling provided no qualitative changes in the propagated wave pattern. We conclude that the results are valid under a certain loss of current to the SMC layer.

In order to simulate the hyperpolarization-based mechanism of conducted vasodilation (Crane et al. 2004), we arranged the individual cell models in a gap junction coupled array and tested whether such a system can support the propagation of hyperpolarizing pulses. We have found, however, that an additional slow adaptation process is needed in order to support the repolarization wave. With inclusion of such a process, our model shows the expected behavior.

The model variable that describes slow adaptation process was introduced for dynamical reasons and needs further physiological justification. Note, however, that the assumptions we used were rather generic and several EC ionic currents as well as feedback responses from the connected SMCs can potentially be suitable in this regard.

As regards time scales, we observed that the propagation speed for both the H- and the R-fronts is about 50 units per 10 ms. Assuming 40 $\upmu$m working length of each EC, i.e., the distance between the gap junctions located near the opposite ends of the cell, we obtain an estimated speed of 20 mm per second, which is consistent with available experimental data. With this, the slow adaptation should have a characteristic time around 5–10 ms, which seems to be realistic.

From a dynamical viewpoint, alternative mechanisms, such as repolarization of an EC by SMC-generated spikes, that provide the capability of the bistable model for propagation of long pulses, can also be suggested. However, we focused here on the slow adaptation mechanism since it seems to be the most relevant and could cover many physiological pathways.

The basic criterion for bistability is an *N*-shaped current–voltage curve with three crossings of the voltage axis (please see Fig. 4 as well as review by Bernd and Droogmans and references herein Nilius and Droogmans (2001). We focused on the potential role of an inwardly rectifying potassium channel in providing the endothelial cells with this particular current–voltage curve. The endothelial cell, however, has numerous other ion channels, pumps, and transporters, and specific combinations of these may equally well provide the cell with this specific property. Also, bistable features is unlikely to be universally present in the microcirculation; in many cases, the current–voltage curve may rather be monotonically increasing leading to a simple decay of the signal along the endothelium (Behringer et al. 2012; Behringer and Segal 2012).

To initiate a dilatation in the bistable case, an initial hyperpolarization is necessary to move the system past the unstable fix-point located where the descending part of the current–voltage curve crosses the voltage axis. What exactly causes this initial small hyperpolarization is unclear, but it may either be a direct influence on the endothelium originating in some form from tissue metabolism and/or this influence may be mediated through the surrounding smooth muscle cell layer. As the threshold is passed (Fig. 4 lower panel), the signal passes without decay along the vascular wall even if some current is lost to the SMC layer. The cells at the wave front provide the initiating small hyperpolarization to their neighbors causing them to switch to the fully hyperpolarized state and so forth. This naturally raises the question as to how the signal is terminated, avoiding it from running into and affecting flow inappropriately in tissue regions where an increase in flow is not needed. To that end, one mechanism may simply be that the signal terminates at branch points where it meets a larger vessel. The larger endothelial mass of the larger vessel may act as a current sink to a degree that prevents passage across the threshold and hence prevents initiation of propagation in the larger vessel. In contrast, if the signal arrives simultaneously from two downstream vessels, it may propagate. Alternatively, the signal may terminate by entering into areas where the endothelium does not show bistable behavior, causing it to decay along the vessel.

Finally, our results showed that a bistability-powered mechanism for regenerative vasodilatory pulse transmission is self-consistent from dynamical viewpoint. Which specific currents, besides the explicitly used in the model background and potassium inwardly rectifying currents, could provide all this? May hyperpolarizing currents such as calcium-sensitive potassium currents play some role? Is there some kind of SMC-mediated positive feedback, supporting the bistability? All these questions are of evident interest for further studies on the topic.

## Footnotes

1 ”one-component units“ refer to one-component reaction–diffusion equations concerning the concentration of a single substance.
